# Large Variations in Risk of Hepatocellular Carcinoma and Mortality in Treatment Naïve Hepatitis B Patients: Systematic Review with Meta-Analyses

**DOI:** 10.1371/journal.pone.0107177

**Published:** 2014-09-16

**Authors:** Maja Thiele, Lise Lotte Gluud, Annette Dam Fialla, Emilie Kirstine Dahl, Aleksander Krag

**Affiliations:** 1 Department of Gastroenterology and Hepatology, Odense University Hospital, Odense, Denmark; 2 Gastrounit, Medical Division, Copenhagen University Hospital, Hvidovre, Denmark; CRCL-INSERM, France

## Abstract

**Background:**

The complications to chronic hepatitis B (HBV) include incidence of hepatocellular carcinoma (HCC) and mortality. The risk of these complications may vary in different patient groups.

**Aim:**

To estimate the incidence and predictors of HCC and in untreated HBV patients.

**Methods:**

Systematic review with random effects meta-analyses of randomized controlled trials and observational studies. Results are expressed as annual incidence (events per 100 person-years) with 95% confidence intervals. Subgroup and sensitivity analyses of patient and study characteristics were performed to identify common risk factors.

**Results:**

We included 68 trials and studies with a total of 27,584 patients (264,919 person-years). In total, 1,285 of 26,687 (5%) patients developed HCC and 730 of 12,511 (6%) patients died. The annual incidence was 0.88 (95% CI, 0.76–0.99) for HCC and 1.26 (95% CI, 1.01–1.51) for mortality. Patients with cirrhosis had a higher risk of HCC (incidence 3.16; 95% CI, 2.58–3.74) than patients without cirrhosis (0.10; 95% CI, 0.02–0.18). The risk of dying was also higher for patients with than patients without cirrhosis (4.89; 95% CI, 3.16–6.63; and 0.11; 95% CI, 0.09–0.14). The risk of developing HCC increased with HCV coinfection, older age and inflammatory activity. The country of origin did not clearly predict HCC or mortality estimates.

**Conclusions:**

Cirrhosis was the strongest predictor of HCC incidence and mortality. Patients with HBV cirrhosis have a 31-fold increased risk of HCC and a 44-fold increased mortality compared to non-cirrhotic patients. The low incidence rates should be taken into account when considering HCC screening in non-cirrhotic patients.

**Trial Registration:**

Prospero CRD42013004764

## Introduction

Chronic hepatitis B virus (HBV) affects several hundred million people worldwide. Complications include hepatocellular carcinoma (HCC) and death from liver failure. Antiviral therapies have improved the management of HBV, but treatment is costly and associated with adverse events. As a result several patients with HBV remain untreated [1].

Guidelines recommend HCC screening in patients with HBV, but the validity of the underlying evidence is questionable. Additionally, screening is expensive and has been difficult to implement [2]. Based on cost-benefit analyses, screening is recommended if the annual incidence exceeds 0.2 per 100 patient-years [3]. Incidence estimates for subgroups can help identify high risk patients that may benefit from screening.

Previous studies have evaluated the prognosis of untreated HBV based on central registries [4], [5]. This design may underestimate event rates due to inaccurate registration. Discrepancies in the results of cohort studies from Asia, Europe and North America [4], [6]–[8] may reflect differences in study populations such as degree of fibrosis, hepatitis B envelope antigen (HBeAg) positivity, gender, age, disease activity, ethnicity, genotypes and coinfections with hepatitis C (HCV), hepatitis D (HDV) and human immunodeficiency virus (HIV) [9]. Unlike central registries, randomised controlled trials (RCTs) and observational studies include standardised registration and follow up. Several trials and studies on HBV include an untreated control group. We therefore conducted a systematic review to evaluate the incidence of HCC and mortality and the potential influence of patient and study characteristics in untreated patients with HBV, based on analyses of RCTs and observational studies.

## Methods

The review is based on a registered protocol (Prospero ID CRD42013004764) and follows the MOOSE guidelines and the Cochrane Handbook for Systematic Reviews [10], [11].

### Data Sources and Searches

Electronic and manual searches were combined (Checklist S1). The last search update was September 2013.

### Study Selection

RCTs and observational studies (prospective cohort and case-control studies) on patients with chronic HBV were eligible for inclusion. Data on untreated patients (patients allocated to placebo or no intervention) were included. The primary outcomes were HCC incidence and all-cause mortality. The secondary outcome was HCC related mortality. To minimize detection and ascertainment bias, studies assembling outcome data from central registries were excluded. All outcomes were assessed after at least 12 months of follow up to exclude prevalent HCC.

### Data Extraction

Three authors independently extracted data (MT, ADF, ED). In case of discrepancies, a fourth author was contacted (AK). Extracted data (Datasheet S1) included characteristics of patients (cirrhosis, gender, age, HBeAg positivity, coinfections, Hepatitis B virus DNA (HBV-DNA), alanine aminotransferase (ALT) and HBV genotype) and trials (design, country of origin, duration of follow up, HCC screening and risk of bias).

### Quality Assessment

The assessment of bias followed the Critical Appraisal Tool from the Center for Evidence Based Medicine [12]. For each study, we evaluated whether patients were assembled at a common point in the course of their disease, whether the follow up data were complete, whether outcome criteria were either objective or applied in a blind fashion, how likely the outcomes were over time and precision of the prognostic estimates.

### Data Synthesis and Analysis

The analyses were performed in Stata version 13 (Statacorp, TX, USA). Incidence estimates (with the corresponding standard errors) were calculated based on the event rates in relation to the duration of follow up (number of person years). Random effects model meta-analyses were performed with results expressed as annual incidence (number of events per 100 person-years) and 95% confidence intervals (CI). I^2^ was calculated as a marker of heterogeneity. I^2^ values above 50% were considered as important heterogeneity.

The subgroup analyses evaluated the influence of risk factors related to patient and trial characteristics (cirrhosis, HBeAg status, coinfections, gender, age, HBV-DNA, ALT, genotype, HCC screening, study design and country of origin). We used Eggers test for funnel plot asymmetry to test for small study effects. We also performed a post-hoc subgroup analyses that combined cirrhosis and country of origin. The analysis estimated the incidence of HCC and mortality of patients with or without cirrhosis stratified by the geographical region (Asia or Europe).

Due to high heterogeneity post-hoc meta-regression analyses were performed to test for study-level covariates. The covariates included in the meta-regression were proportion with cirrhosis, HBeAg positivity, male gender, mean age, proportion with elevated HBV-DNA, proportion with elevated ALT, HCC screening, study design and study region. Post-hoc investigations on the influence of each individual study on the results of meta-analyses were also performed.

## Results

### Search Results and Characteristics of Included Studies

**Figure 1:**
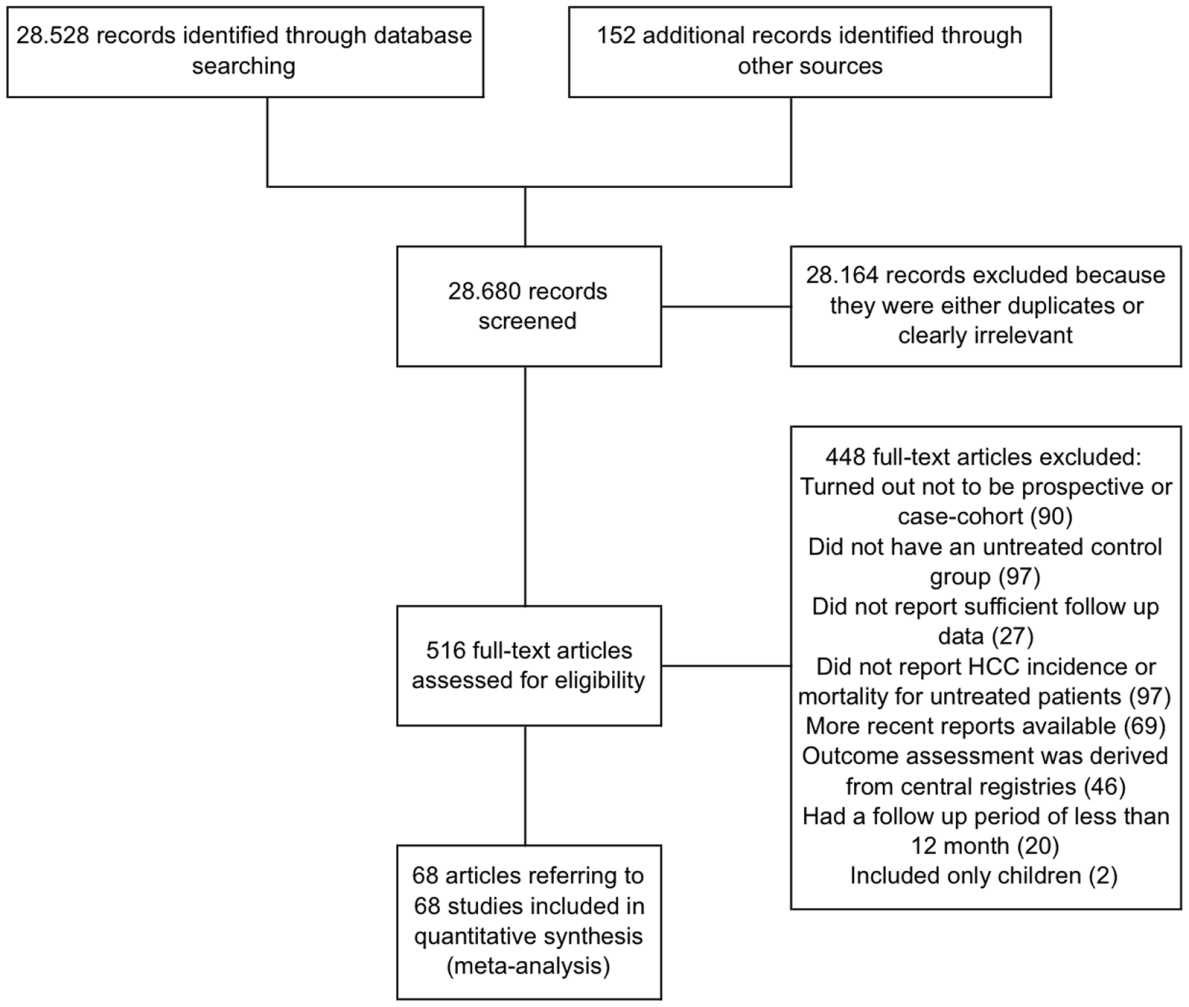
Trial flow diagram.

The initial searches identified 28,680 potentially eligible references (Figure 1). Sixty-eight of these references referred to studies that fulfilled our inclusion criteria (7 RCTs [13]–[19], 49 prospective cohort studies [20]–[68] and 12 case-control studies [69]–[80]).

HCC screening was performed in 50 studies (three RCTs and 43 observational studies). The quality of the included studies was high with regards to objectiveness of the outcome criteria and the likelihood of outcomes over time. The precision of the outcome estimate was low with a standard deviation of the mean follow up period above 50% in fifteen studies and a standard deviation of 25–50% in 30 studies. Most studies included patients at different time points in the course of the disease. The completeness of follow-up data was sufficiently reported in 13 studies (Table 1).

**Table 1:** Bias assessment.

Bias domains	Number of trials with low risk of bias	Number of trials with uncertain risk of bias	Number of trials with high risk of bias
Were patients assembled at a common point in the course of their disease?^1^	9	17	42
Were the follow up data complete?^2^	13	55	0
Were outcome criteria either objective or applied in a blind fashion?^3^	68	0	0
How likely are the outcomes over time?^4^	68	0	0
How precise are the prognostic estimates?^5^	15	30	23

1 Low risk of bias if all patients are assembled at a common time point in the course of their disease. Unknown risk of bias if relevant information for assessment of bias can not be assembled. High risk of bias if patients are assembled at different time points in the course of their disease.

2 Low risk of bias if all patients are acounted for and losses to follow up not likely to affect the outcome estimate. Uncertain risk of bias if data on losses to follow up are missing/not accounted for. High risk of bias if losses to follow up are likely to affect outcome estimate.

3 All trials low risk of bias as HCC and/or mortality are objective outcome measures.

4 All trials low risk of bias as the review only included studies with adequate follow up period (>1 year).

5 Low risk of bias if standard deviation of follow up <25% of the mean follow up. Uncertain risk of bias if standard deviation of follow up is 25–50% of the mean follow up. High risk of bias is standard deviation of follow up is >50% of the mean follow up.

### Characteristics of Included Patients

Our analyses included 27,584 patients and 264,919 person-years. The minimum duration of follow up was 2.0 years and the maximum duration of follow up was 16.5 years (Table S1). The studies were performed in Asia (23,537 patients; *n* = 35 studies), Europe (2,401 patients; *n* = 29 studies) and North America (1,646 patients, *n* = 4 studies). Disease severity ranged from asymptomatic carriers to decompensated cirrhosis. In twenty-eight studies all included patients had cirrhosis at baseline. In 11 studies, all included patients were non-cirrhotic. In the studies that reported the proportion of patients with cirrhosis at inclusion, the median proportion of patients with cirrhosis at baseline was 41%. In total, 3,382 of 23,097 patients (15%) were cirrhotic. Outcome data was available for 3,673 patients with cirrhosis and 16,949 patients without cirrhosis (Table S2). For the remainder of patients, data on cirrhosis status could not be extracted. Most patients were men (range 24–100%). The mean age ranged from 26 to 65 years. Six studies only included HBeAg-positive patients and 11 only included HBeAg-negative patients. In total, 726 of 1,290 (56%) patients spontaneously cleared HBeAg during the study. HCV coinfection was present in 326 patients (*n* = 14 studies) and 437 had HDV coinfection (*n* = 10 studies). None of the studies included HIV-positive patients.

### Incidence of HCC

**Figure 2:**
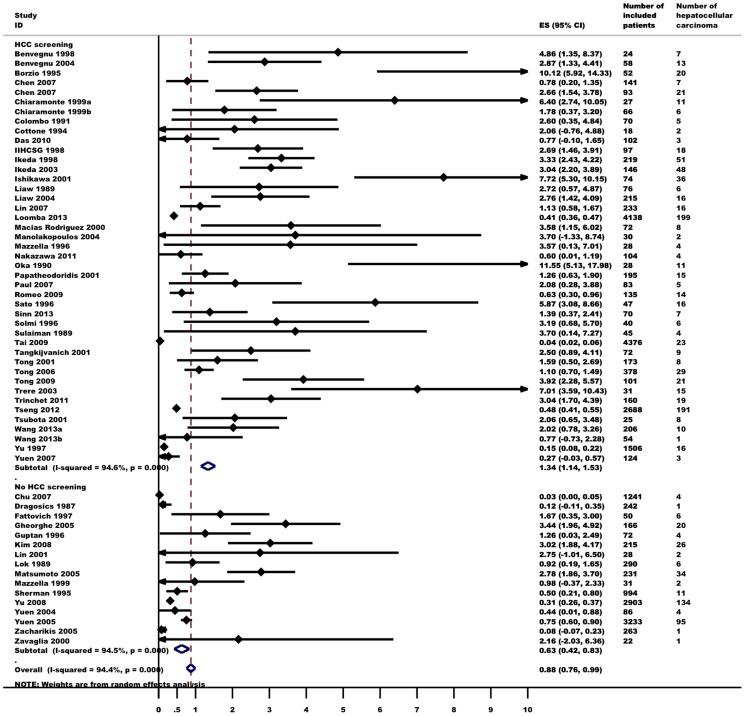
Annual incidence of hepatocellular carcinoma in untreated hepatitis B patients (events per 100 person-year). Random effects meta-analysis with subgroups according to HCC screening.

**Figure 3:**
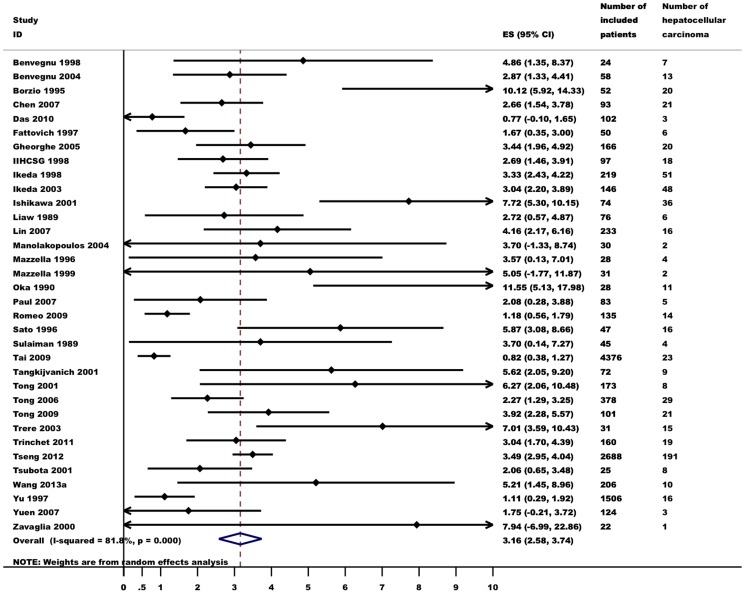
Annual incidence of hepatocellular carcinoma in untreated hepatitis B patients with cirrhosis (events per 100 person-year). Random effects meta-analysis.

Data on the incidence of HCC were gathered from 57 studies. HCC was diagnosed in 1,285 of 26,687 patients (5%). Random effects meta-analysis showed that the annual incidence of HCC was 0.88 (95% CI 0.76–0.99, I^2^ = 94%, Figure 2) per 100 person-years. The incidence of HCC varied considerably in different subgroups (Table 2). In 33 studies, 605 of 2,660 patients (23%) with cirrhosis developed HCC (Figure 3). Five studies found that 71 of 8,471 patients (1%) without cirrhosis developed HCC. In subgroup meta-analyses, the incidence of HCC among patients with cirrhosis was 3.16 (95% CI 2.58–3.74, I^2^ = 82%), which was 31-fold higher than among patients without cirrhosis (0.10, 95% CI 0.02–0.18, I^2^ = 90%). HCV coinfection (reported in 9 studies) lead to a 4-fold increase in the risk of HCC (3.73, 95% CI 1.59–5.86, I^2^ = 78%). Age and the proportion of patients with elevated ALT also predicted the incidence of HCC. Gender, HBeAg-status and HBV-DNA did not seem to be related to HCC incidence. The data did not allow for an assessment of the influence of HDV coinfection or genotype.

**Table 2:** Annual Hepatocellular Carcinoma Incidence and Mortality Rates in Chronic Hepatitis B Patients.

Variable	HCC incidence^1^	95% CI	Mortality^1^	95% CI
Overall	0.88	0.76–0.99	1.26	1.01–1.51
Cirrhosis	3.16	2.58–3.74	4.89	3.16–6.63
Non-cirrhosis	0.10	0.02–0.18	0.11	0.09–0.14
HCC mortality	NA		0.34	0.22–0.45
HBeAg positive	1.47	0.40–2.55	NA	
HBeAg negative	0.72	0.21–1.23	NA	
HCV coinfection	3.73	1.59–5.86	NA	
Male	0.63	0.40–0.86	NA	
Female	0.29	0.04–0.53	NA	
Mean age >50 years	3.92	2.72–5.11	NA	
Mean age <50 years	0.82	0.69–0.95	1.69	1.28–2.10
Elevated HBV-DNA^2^	1.50	1.12–1.88	2.37	1.18–3.56
No HBV-DNA^3^	2.14	0.04–4.25	2.90	1.32–4.49
Elevated ALT^2^	1.86	1.30–2.42	2.78	1.51–4.05
Normal ALT^3^	0.32	0.21–0.43	0.30	0.12–0.49
HCC screening	1.34	1.14–1.53	1.48	1.11–1.84
No HCC screening	0.63	0.42–0.83	1.50	0.98–2.01
Randomized trials	1.95	1.16–2.75	1.57	0.00–3.53
Prospective cohorts	0.76	0.63–0.88	1.15	0.90–1.39
Case-control series	1.30	0.81–1.79	7.30	2.22–12.38
Asia	0.75	0.62–0.88	0.91	0.61–1.20
Europe	2.09	1.56–2.62	2.49	1.60–3.39
North America	1.41	0.60–2.22	NA	

1 Number of HCC or deaths per 100 person-year. Meta-analysis using a random effects model with incidence as the study effect size and standard error of the incidence as study effect variation.

2 Studies including >50% of patients with elevated HBV-DNA/ALT.

3 Studies including >50% of patients with unmeasurable HBV-DNA/normal ALT.

ALT, alanine aminotransferase; CI, confidence interval; HBeAg, hepatitis B envelope antigen; HBV-DNA, hepatitis B virus DNA; HCC, hepatocellular carcinoma; HCV, chronic hepatitis C; NA, not analyzed/not enough data for analysis.

The incidence of HCC was higher in studies with systematic HCC screening (1.34, 95% CI 1.14–1.53) than in studies without screening (0.63, 95% CI 0.42–0.83). The annual incidence of HCC was 1.95 (95% CI 1.16–2.75) in RCTs, 0.76 (95% CI 0.63–0.88) in prospective cohorts and 1.30 (95% CI 0.81–1.79) in case-control series.

European studies had a three-fold higher annual incidence than Asian studies (2.09, 95% CI 1.56–2.62 and 0.75, 95% CI 0.62–0.88, respectively). The median proportion of patients with cirrhosis at inclusion was 30% for Asian studies compared to 100% for European studies. All patients had cirrhosis at baseline in 16 of 29 European studies (55%) and only 12 of 35 Asian studies (34%). The HCC incidence in patients with cirrhosis did not differ between the two regions (Europe 3.35, 95% CI 2.31–4.39; Asia 3.06, 95% CI 2.24–3.87). There was insufficient data to assess HCC in European non-cirrhotic patients.

In the primary meta-analysis and most of the subgroup analyses (except RCTs and in studies including a high proportion of patients with elevated ALT) we found evidence of heterogeneity (I^2^>50%). Evidence of small study effects (Egger's test *P*<0.05) was found in all subgroups except for patients without cirrhosis, females, HBe-Ag negative patients, studies including patients with low HBV-DNA, studies including a high proportion of patients with elevated ALT, RCTs and studies performed in North America).

Univariate metaregression analyses of study-level covariates showed that the overall annual incidence estimate was predicted by proportion with cirrhosis (coefficient  = 0.024, *P*<0.001), mean age (coefficient  = 0.100, *P* = 0.001), proportion with inflammatory activity (coefficient  = 0.014, *P* = 0.013) and whether HCC screening was performed (coefficient  = 1.050, *P* = 0.037). None of the variables were significant predictors of the incidence estimate in multivariate metaregression. Analysing the effect of each individual study on the overall incidence estimate revealed that exclusion of either one of two studies would increase the estimate outside its 95% upper CI (Figure S1). Both were prospective cohort studies on several thousand asymptomatic HBsAg carriers [54], [64].

### Mortality

**Figure 4:**
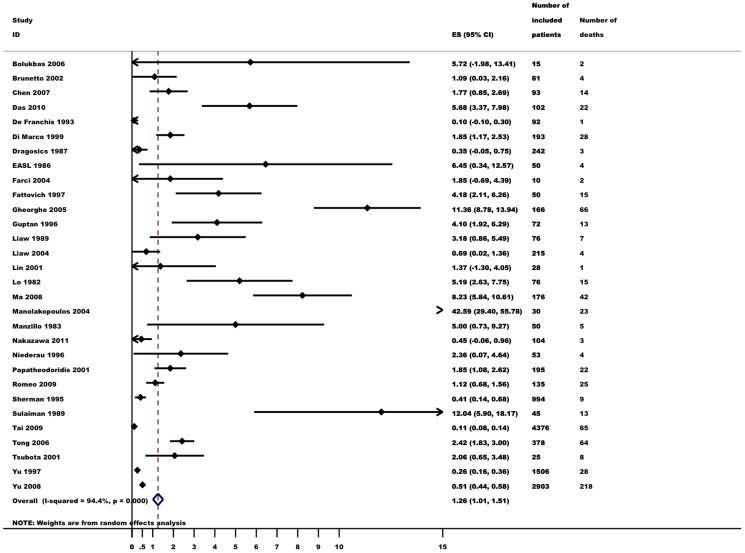
Annual mortality (events per 100 person-year) in untreated hepatitis B patients. Random effects meta-analysis.

Thirty studies reported mortality. In total, 730 of 12,511 patients (6%) died (Table 2). Random effects meta-analysis showed that the risk of death (annual mortality per 100 person years) was 1.26 (95% CI 1.01–1.51, I^2^ = 94%, Figure 4). Twelve studies reported mortality in patients with cirrhosis, in which 266 of 951 patients (28%) with cirrhosis died. Three studies on patients without cirrhosis reported that 69 of 4,572 died (2%). Subgroup analyses confirmed that patients with cirrhosis had a higher mortality (4.89, 95% CI 3.16–6.63, I^2^ = 91%) than patients without cirrhosis (0.11, 95% CI 0.09–0.14, I^2^ = 0%). Mortality was higher in studies including a high proportion of patients with inflammatory activity (2.78, 95% CI 1.51–4.05) than in studies with a low proportion of these patients (0.30, 95% CI 0.12–0.49). The mortality did not appear to depend on the proportion of included patients with elevated HBV-DNA.

HCC screening had no influence on the annual mortality per 100 person years. Mortality was higher in case-control series (7.30, 95% CI 2.22–12.38) than in prospective cohorts (1.15, 95% CI 0.90–1.39), but not RCTs (1.57, 95% CI 0.00–3.53). The very high mortality in case-control series was largely carried by a study on patients with decompensated cirrhosis [74]. European studies had higher mortality (2.49, 95% CI 1.60–3.39) than Asian studies (0.91, 95% CI 0.61–1.20). The geographical heterogeneity was evaluated in a post-hoc analysis considering the higher proportion of patients with cirrhosis at inclusion in European studies. Although mortality in patients with cirrhosis seemed higher in European than in Asian studies (9.45, 95% CI 4.11–14.78 versus 3.17, 95% CI 1.59–4.74) the difference in mortality was not significant (test for subgroup differences P>0.05). The high mortality in European studies was primarily due to a study on patients with decompensated cirrhosis and elevated transaminases [74]. There was not enough data on mortality in European non-cirrhotic patients to allow for a post-hoc analysis on differences in non-cirrhotic mortality.

In all subgroup analyses except patients without cirrhosis there was evidence of heterogeneity with I^2^ values above 50%. Evidence of small study effects (Egger's test *P*<0.05) was present in most analyses except trials including a high proportion of patients with normal ALT, trials including a high proportion of patients with low HBV-DNA and RCTs.

Analysing the effect of each individual study on the overall mortality estimate revealed that the estimate would increase beyond its 95% upper CI if either one of two large studies on asymptomatic carriers were excluded (Figure S2) [49], [64].

Twenty-one studies reported HCC related mortality (190 of 7,641 patients; 3%). The risk of HCC related mortality was 0.34 per 100 person years (95% CI 0.22–0.45). There was considerable heterogeneity (I^2^ = 74%) and clear evidence of small study effects (Egger's test *P*<0.001).

## Discussion

This systematic review includes data from 68 studies and more than 27,000 patients. Overall, the risk of developing HCC and mortality was high. The large number of patients and studies allowed for adequate statistical power in subgroup analyses. The subgroup analyses led to the identification of several high risk groups. In particular, the risk of HCC was 31-fold higher if patients had cirrhosis. Likewise, cirrhosis was associated with a 44-fold increase in mortality. Inflammatory activity also predicted a higher risk of HCC and mortality. The risk of HCC was higher if HCC screening was applied suggesting that cases of HCC may have been overlooked in studies without systematic screening. Other predictors of a higher risk of HCC included HCV coinfection and older age.

Guidelines recommend HCC screening in patients with HBV. However, screening is expensive and implementation is difficult. The identification of high risk groups may help identify the groups with the highest benefit of screening. Based on cost-benefit analyses, screening is recommended if the annual incidence exceeds 0.2 per 100 person-years [3]. In our overall analysis, the annual incidence of HCC was 0.88 per 100 person years. For patients without cirrhosis, the incidence was only 0.10. Accordingly, cost of screening HBV patients without cirrhosis may outweigh the benefits. Additionally, it is important to balance potential benefits with potential harms of screening [81]. Our results indicate that the lowest HCC incidence can be found in young, asymptomatic carriers without hepatic inflammation. The negative consequences (such as false positive findings) of screening may be too high for this patient group [82]. It was however not within the scope of the review to suggest a prognostic algorithm for HCC or mortality including several risk factors, as this would only be possible from a meta-analysis with individual patient data.

Improved detection of HCC does not guarantee reduced cancer specific mortality. In our analysis, despite that studies with systematic HCC screening had a higher HCC incidence than studies without screening, screening studies did not have lower mortality. Our results do however not allow for an analysis of the impact of HCC screening on patient harm, costs or mortality.

In agreement with previous evidence, we found that the level of inflammation is an important predictor of clinical outcomes in HBV [83], [84]. We were able to analyse the influence of cirrhosis, HBeAg status and HCV coinfection, gender, age and markers of disease activity, but not other known predictors such as HDV and HIV coinfection, genotype, alcohol, diabetes or smoking [85], [86]. Additional evidence is needed to determine the influence of these factors.

In most analyses we found evidence of small study effects and high heterogeneity. One of the reasons for this finding is that small studies included a high proportion of patients with cirrhosis. The larger studies mostly included asymptomatic HBsAg carriers. Additionally, our bias assessment revealed that most studies included patients at different time points in the disease, leading to heterogeneous patient populations. Likewise, the heterogeneity of the included studies may be a reflection of the heterogeneous population of chronic HBV infected patients worldwide, thus making statistical heterogeneity difficult to avoid.

We found considerable geographical differences in incidence of HCC and mortality. These differences were caused by a much larger number of European studies including only patients with cirrhosis, compared to Asian studies. However, HCC incidence and mortality in patients with cirrhosis was similar in European and Asian studies, thus supporting our conclusion that cirrhosis was the strongest predictor of HCC and mortality. One may speculate whether the large number studies including only patients with cirrhosis would skew the results of our analyses towards a higher overall HCC incidence and mortality. However, since the studies with the largest number of included patients were mostly in non-cirrhotic patients, only 15% of the total study population had cirrhosis at baseline.

In conclusion, the combined evidence stresses the importance of risk stratification in HBV. The HCC incidence and mortality depends on a number of patient characteristics. In non-cirrhotic patients without inflammatory activity HCC screening could be futile due to the low incidence, whereas efforts should be made to detect HCC in at-risk patients with cirrhosis, HCV coinfection, old age and inflammatory activity.

## Supporting Information

Figure S1 **Analysis of the influence of a single study on the overall estimate in the meta-analysis, HCC incidence.**
(TIFF)

Figure S2 **Analysis of the influence of a single study on the overall estimate in the meta-analysis, mortality.**
(TIFF)

Table S1 **Supplementary table, trial characteristics.**
(DOCX)

Table S2 **Supplementary table, HCC incidence and mortality in included studies.**
(DOCX)

Checklist S1 **MOOSE checklist.**
(DOCX)

Datasheet S1 **Data extraction sheet.**
(DTA)
