# Supplementary figures and images for: Large Variations in Risk of Hepatocellular Carcinoma and Mortality in Treatment Naïve Hepatitis B Patients: Systematic Review with Meta-Analyses
Source: PLoS One. 2014 Sep 16;9(9):e107177. doi: 10.1371/journal.pone.0107177 (PMC4167336; doi:10.1371/journal.pone.0107177)

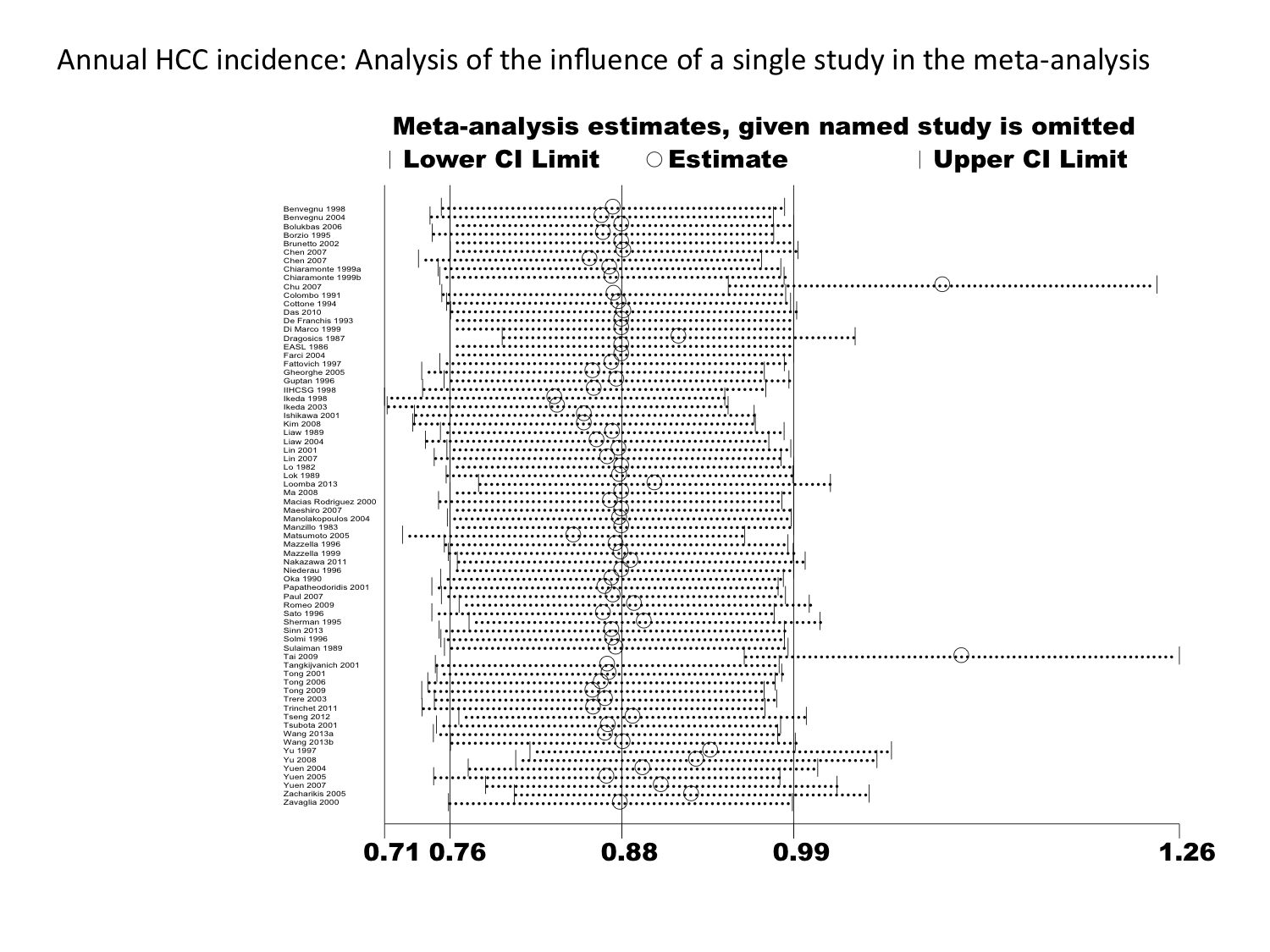

Supplement: Figure S1 — Analysis of the influence of a single study on the overall estimate in the meta-analysis, HCC incidence. (TIFF) [file pone.0107177.s001.tiff]

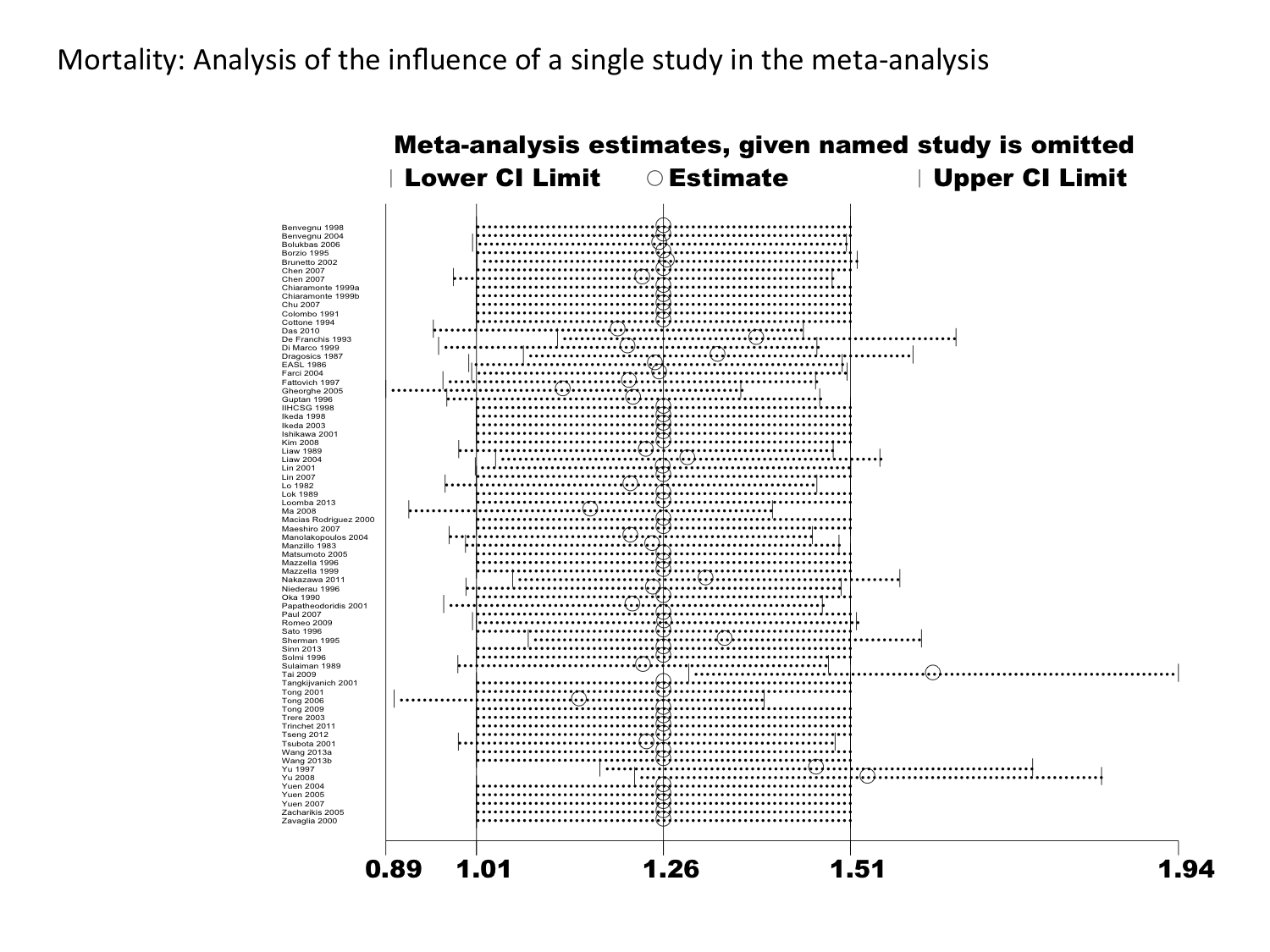

Supplement: Figure S2 — Analysis of the influence of a single study on the overall estimate in the meta-analysis, mortality. (TIFF) [file pone.0107177.s002.tiff]
