# Supplementary material for: Large Variations in Risk of Hepatocellular Carcinoma and Mortality in Treatment Naïve Hepatitis B Patients: Systematic Review with Meta-Analyses
Source: PLoS One. 2014 Sep 16;9(9):e107177. doi: 10.1371/journal.pone.0107177 (PMC4167336; doi:10.1371/journal.pone.0107177)
Supplement: Table S1 — Supplementary table, trial characteristics. (DOCX) [file pone.0107177.s003.docx]

| Supporting table S3*.* Characteristics of included studies | | | | |
| --- | --- | --- | --- | --- |
| Study, year | Country of origin | Number of patients | Follow up (mean±SD, years) | HCC screening |
| Randomized trials: | | | | |
| EASL 198 | Europe | 50 | 1.2±0.7 | NS |
| Farci 2004 | Italy | 10 | 10.8±3.8 | NS |
| Liaw 2004 | Asia | 215 | 2.7±0.6 | Yes |
| Mazzella 1999 | Italy | 31 | 6.6±0.6 | NS |
| Trinchet 2011 | France | 160 | 3.9±0.5 | Yes |
| Wang 2013 | China | 260 | 2.4±1.0 | Yes |
| Zavaglia 2000 | Italy | 22 | 2.1±0.4 | NS |
| Prospective cohorts: | | | | |
| Benvegnu 1998 | Italy | 24 | 6.0±2.2 | Yes |
| Benvegnu 2004 | Italy | 58 | 7.8±3.8 | Yes |
| Borzio 1995 | Italy | 52 | 3.8±1.9 | Yes |
| Brunetto 2002 | Italy | 61 | 6.0±2.3 | Yes |
| Chen 2007 | China | 141 | 6.4±2.0 | Yes |
| Chen 2007 | China | 93 | 8.5±5.0 | Yes |
| Chiaramonte 1999 | Italy | 93 | 5.1±2.8 | Yes |
| Chu 2007 | Chian | 1241 | 12.3±5.5 | NS |
| Colombo 1991 | Italy | 70 | 2.8±0.8 | Yes |
| Cottone 1994 | Italy | 18 | 5.4±1.8 | Yes |
| De Franchis 1993 | Italy | 92 | 10.8±1.4 | NS |
| Di Marco 1999 | Italy | 193 | 7.8±3.1 | Yes |
| Dragosics 1987 | Austria | 242 | 3.5±1.9 | NS |
| Fattovich 1997 | Europe | 50 | 7.2±5.1 | NS |
| Gheorghe 2005 | Romania | 166 | 3.5±2.5 | NS |
| Guptan 1996 | Asia-India | 72 | 4.4±2.1 | NS |
| Ikeda 1998 | Japan | 219 | 7.0±3.7 | Yes |
| Ikeda 2003 | Japan | 146 | 10.8±3.1 | Yes |
| Ishikawa 2001 | Japan | 74 | 6.3±2.4 | Yes |
| Kim 2008 | Korea | 215 | 4.0±0.8 | NS |
| Liaw 1989 | China | 76 | 2.9±1.8 | Yes |
| Lo 1982 | China | 76 | 3.8±0.9 | NS |
| Lok 1989 | China | 290 | 2.3±0.5 | NS |
| Loomba 2013 | China | 4138 | 11.6±1.7 | Yes |
| Ma 2005 | China | 176 | 2.9±0.6 | Yes |
| Macias Rodriguez 2000 | Spain | 72 | 3.1±1.3 | Yes |
| Maeshiro 2007 | Japan | 121 | 6.7±4.0 | Yes |
| Manzillo 1983 | Italy | 50 | 2.0±0.6 | NS |
| Mazzella 199 | Italy | 28 | 4.0±0.4 | Yes |
| Nakazawa 2011 | Japan | 104 | 6.4±3.4 | Yes |
| Oka 1990 | Japan | 28 | 3.4±1.5 | Yes |
| Papatheodoridis 200 | Greece | 195 | 6.1±3.9 | Yes |
| Paul 2007 | India | 83 | 2.9±2.1 | Yes |
| Romeo 2009 | Italy | 135 | 16.5±7.1 | Yes |
| Sato 1996 | Japan | 47 | 5.8±3.8 | Yes |
| Sherman 1995 | North America | 994 | 2.2±0.8 | NS |
| Solmi 1996 | Italy | 40 | 4.7±1.1 | Yes |
| Sulaiman 1989 | Indonesia | 45 | 2.4±1.3 | Yes |
| Tai 2009 | China | 4376 | 13.4±5.2 | Yes |
| Tong 2001 | USA | 173 | 2.9±2.0 | Yes |
| Tong 2006 | USA | 378 | 7.0±3.3 | Yes |
| Tong 2009 | USA | 101 | 5.3±3.1 | Yes |
| Trere 2003 | Italy | 31 | 6.9±4.4 | Yes |
| Tsubota 2001 | Japan | 25 | 15.5±7.3 | Yes |
| Yu 1997 | China | 1506 | 7.1±2.8 | Yes |
| Yu 2008 | China | 2903 | 14.7±2.5 | NS |
| Yuen 2004 | China | 86 | 10.5±3.5 | NS |
| Yuen 2005 | China | 3233 | 3.9±3.9 | NS |
| Zacharikis 2005 | Greece | 263 | 4.9±1.7 | NS |
| Case control series: | | | | |
| Bolukbas 2006 | Turkey | 15 | 2.0±1.1 | Yes |
| Das 2010 | India | 102 | 3.8±4.3 | Yes |
| IIHCSG 1998 | Italy and Argentina | 97 | 6.9±3.3 | Yes |
| Lin 2001 | China | 28 | 2.6±1.1 | NS |
| Lin 2007 | China | 233 | 6.1±3.0 | Yes |
| Manolakopoulos 2004 | Greece | 30 | 1.8±1.1 | Yes |
| Matsumoto 2005 | Japan | 231 | 5.3±4.7 | NS |
| Niederau 1996 | Germany | 53 | 3.2±1.5 | NS |
| Sinn 2013 | Korea | 70 | 7.2±1.7 | Yes |
| Tangkijvanich 2001 | Thailand | 72 | 5.0±2.3 | Yes |
| Tseng 2012 | China | 2688 | 14.7±4.3 | Yes |
| Yuen 2007 | China | 124 | 9.0±1.3 | Yes |
| SD, standard deviation; HCC, hepatocellular carcinoma; NS, not stated | | | | |
