# Supplementary material for: Large Variations in Risk of Hepatocellular Carcinoma and Mortality in Treatment Naïve Hepatitis B Patients: Systematic Review with Meta-Analyses
Source: PLoS One. 2014 Sep 16;9(9):e107177. doi: 10.1371/journal.pone.0107177 (PMC4167336; doi:10.1371/journal.pone.0107177)
Supplement: Table S2 — Supplementary table, HCC incidence and mortality in included studies. (DOCX) [file pone.0107177.s004.docx]

| **Supporting table S4*.*** HCC incidence and mortality in included studies | | | | | |
| --- | --- | --- | --- | --- | --- |
| Study, year | Patients | Developed HCC | Died | HCC incidence | Mortality |
| Randomized trials: | | | | | |
| EASL 1986 | 50 |  | 4 |  | 6.45 |
| Farci 2004 | 10 |  | 2 |  | 1.85 |
| Liaw 2004 | 215 | 16 | 4 | 2.76 | 0.69 |
| Mazzella 1999 | 31 | 2 |  | 0.98 |  |
| Trinchet 2011 | 160 | 19 |  | 3.04 |  |
| Wang 2013 | 260 | 11 |  | 1.76 |  |
| Zavaglia 2000 | 22 | 1 |  | 2.16 |  |
| Prospective cohorts: | | | | | |
| Benvegnu 1998 | 24 | 7 |  | 4.86 |  |
| Benvegnu 2004 | 58 | 13 |  | 2.87 |  |
| Borzio 1995 | 52 | 20 |  | 10.12 |  |
| Brunetto 2002 | 61 |  | 4 |  | 1.09 |
| Chen 2007 | 63 | 21 | 14 | 2.66 | 1.77 |
| Chen 2007 | 141 | 7 |  | 0.78 |  |
| Chiaramonte 1999 | 27 | 11 |  | 6.40 |  |
| Chu 2007 | 66 | 6 |  | 1.78 |  |
| Colombo 1991 | 70 | 5 |  | 2.60 |  |
| Cottone 1994 | 18 | 2 |  | 2.06 |  |
| De Franchis 1993 | 92 |  | 1 |  | 0.10 |
| Di Marco 1999 | 193 |  |  | 28 | 1.85 |
| Dragosics 1987 | 242 | 1 | 3 | 0.12 | 0.35 |
| Fattovich 1997 | 102 | 3 | 22 | 0.77 | 5.68 |
| Gheorghe 2005 | 50 | 6 | 15 | 1.67 | 4.18 |
| Guptan 1996 | 72 | 4 | 13 | 1.26 | 4.10 |
| Ikeda 1998 | 97 | 18 |  | 2.69 |  |
| Ikeda 2003 | 219 | 51 |  | 3.33 |  |
| Ishikawa 2001 | 146 | 48 |  | 3.04 |  |
| Kim 2008 | 215 | 26 |  | 3.02 |  |
| Liaw 1989 | 76 | 6 | 7 | 2.72 | 3.18 |
| Lo 1982 | 76 |  | 15 |  | 5.19 |
| Lok 1989 | 290 | 6 |  | 0.92 |  |
| Loomba 2013 | 4138 | 199 |  | 0.41 |  |
| Ma 2008 | 176 |  | 42 |  | 8.23 |
| M.-Rodriguez 2000 | 233 | 16 |  | 1.13 |  |
| Maeshiro 2007 | 72 | 8 |  | 3.58 |  |
| Manzillo 1983 | 50 |  | 5 |  | 5.00 |
| Mazzella 1996 | 28 | 4 |  | 3.57 |  |
| Nakazawa 2011 | 104 | 4 | 3 | 0.60 | 0.45 |
| Oka 1990 | 28 | 11 |  | 11.55 |  |
| Papatheodoridis 2001 | 195 | 15 | 22 | 1.26 | 1.85 |
| Paul 2007 | 83 | 5 |  | 2.08 |  |
| Romeo 2009 | 53 |  | 4 |  | 2.36 |
| Sato 1996 | 135 | 14 | 25 | 0.63 | 1.12 |
| Sherman 1995 | 994 | 11 | 9 | 0.50 | 0.41 |
| Solmi 1996 | 40 | 6 |  | 3.19 |  |
| Sulaiman 1989 | 45 | 4 | 13 | 3.70 | 12.04 |
| Tai 2009 | 70 | 7 |  | 1.39 |  |
| Tong 2001 | 173 | 8 |  | 1.59 |  |
| Tong 2006 | 378 | 29 | 64 | 1.10 | 2.42 |
| Tong 2009 | 2688 | 191 |  | 0.48 |  |
| Tsubota 2001 | 25 | 8 | 8 | 2.06 | 2.06 |
| Trere 2003 | 101 | 21 |  | 3.92 |  |
| Yu 1997 | 1506 | 16 | 28 | 0.15 | 0.26 |
| Yu 2008 | 2903 | 134 | 218 | 0.31 | 0.51 |
| Yuen 2004 | 31 | 15 |  | 7.01 |  |
| Yuen 2005 | 86 | 4 |  | 0.44 |  |
| Zacharikis 2005 | 263 | 1 |  | 0.08 |  |
| Case control series: | | | | | |
| Bolukbas 2006 | 15 |  | 2 |  | 5.72 |
| Das 2010 | 1241 | 4 |  | 0.03 |  |
| IIHCSG 1998 | 166 | 20 | 66 | 3.44 | 11.36 |
| Lin 2001 | 74 | 36 |  | 7.72 |  |
| Lin 2007 | 28 | 2 | 1 | 2.75 | 1.37 |
| Manolakopoulos 2004 | 121 |  |  |  |  |
| Matsumoto 2005 | 30 | 2 | 23 | 3.70 | 42.59 |
| Niederau 1996 | 231 | 34 |  | 2.78 |  |
| Sinn 2013 | 47 | 16 |  | 5.87 |  |
| Tangkijvanich 2001 | 4376 | 23 | 65 | 0.04 | 0.11 |
| Teng 2012 | 72 | 9 |  | 2.50 |  |
| Yuen 2007 | 3233 | 95 |  | 0.75 |  |
| HCC, hepatocellular carcinoma. | | | | | |
