# Supplementary material for: Large Variations in Risk of Hepatocellular Carcinoma and Mortality in Treatment Naïve Hepatitis B Patients: Systematic Review with Meta-Analyses
Source: PLoS One. 2014 Sep 16;9(9):e107177. doi: 10.1371/journal.pone.0107177 (PMC4167336; doi:10.1371/journal.pone.0107177)
Supplement: Checklist S1 — MOOSE checklist. (DOCX) [file pone.0107177.s005.docx]

Checklist S1: **MOOSE Checklist**

## LARGE VARIATIONS IN RISK OF HEPATOCELLULAR CARCINOMA AND MORTALITY IN TREATMENT NAÏVE HEPATITIS B PATIENTS: META-ANALYSES OF 68 TRIALS AND 27.584 PATIENTS

Corresponding Author:

Maja Thiele, M.D.

Abbreviations:

HBV: Chronic Hepatitis B

HCC: Hepatocellular carcinoma

| **Criteria** | | **Brief description of how the criteria were handled in the meta-analysis** |
| --- | --- | --- |
| **Reporting of background should include** | |  |
| √ | Problem definition | Patients with HBV have an increased risk of HCC and death. Incidence rates according to common risk factors are essential in order to assess prognosis, indication for treatment and HCC surveillance. |
| √ | Hypothesis statement | The incidence of HCC and mortality in HBV differs according in subgroups of patients. |
| √ | Description of study outcomes | Primary outcomes: annual HCC incidence and all-cause mortality.  Secondary outcome: Annual HCC related mortality. |
| √ | Type of exposure or intervention used | Exposure: Chronic hepatitis B. HBV is defined as sustained hepatitis B surface antigen (HBsAg) positivity for more than six months.  Interventions: None  Control: No treatment or placebo, as only treatment naïve patients are included. |
| √ | Type of study designs used | Case control studies, prospective cohorts, randomized trials. |
| √ | Study population | Patients with HBV |
| **Reporting of search strategy should include** | |  |
| √ | Qualifications of searches | The searches were performed in several electronic databases and combined with manual searches. |
| √ | Search strategy, including time period included in the synthesis and keywords | No restriction on time period.  Keywords for HCC were: HCC, hepatoma, hepatocell*, liver cancer, liver neoplasm*, liver cell carcinoma.  Keywords for HBV were: HBV, CHB, hepatitis B, B hepatitis, dane particle, *HBs*, *HBe*, chronic hepatitis B, chronic B hepatitis. |
| √ | Databases and registries searched | PubMed, EMBASE, Science Citation Index Expanded, Cochrane Central Register of Controlled Trials |
| √ | Search software used, name and version, including special features | EndNote was used to manage retrieved citations. |
| √ | Use of hand searching | Included articles and relevant reviews were manually searched for additional eligible articles. |
| √ | List of citations located and those excluded, including justifications | Details of the literature search process are outlined in the flow chart. A list of excluded articles is available upon request. |
| √ | Method of addressing articles published in languages other than English | All articles eligible for inclusion were published in English. |
| √ | Method of handling abstracts and unpublished studies | All trials and studies eligible for inclusion were published as full paper articles. |
| √ | Description of any contact with authors | When necessary authors of eligible trials were contacted in order to provide additional information. |
| **Reporting of methods should include** | |  |
| √ | Description of relevance or appropriateness of studies assembled for assessing the hypothesis to be tested | Inclusion and exclusion criteria are described in the methods section. |
| √ | Rationale for the selection and coding of data | Data on patient characteristics at inclusion and during follow up were selected based on the prognostic factors in HBV and known risk factors for HCC.  Extracted data included:   - Country of origin - Duration of follow up (mean or median) and the variance (SD, SE and/or range) - Whether patients were clearly stated as treatment naïve - Whether HCC screening was performed - Proportion of men and number of men and women - Proportion of patients with histological or clinical cirrhosis and number of patients with cirrhosis at follow up - Severity of underlying liver disease - Proportion with concurrent alcohol abuse - Distribution of genotypes in included patients - Number of patients with coinfections (hepatitis C, hepatitis D, HIV) - Proportion and number of hepatitis envelope antigen (HBeAg) positive patients at inclusion and number of serological response (HBeAg- and HBsAg-seroconversions) during follow up - Proportion of patients with elevated hepatitis B virus DNA (HBV DNA)) - Proportion of patients with elevated alanine aminotransferase (ALT) |
| √ | Assessment of confounding | Sensitivity analyses are described in the methods section.  All analyses were confirmed using fixed effect models. The results are only reported if the overall conclusions differ. |
| √ | Assessment of study quality, including blinding of quality assessors; stratification or regression on possible predictors of study results | Bias assessment are described in the methods section. |
| √ | Assessment of heterogeneity | Assessment of heterogeneity is described in the methods section.  I^2^ values above 50% were considered as a sign of heterogeneity.  Covariates included in the post-hoc meta-regression were proportion with cirrhosis, HBeAg positivity, male gender, mean age, proportion with elevated HBV-DNA, proportion with elevated ALT, HCC screening, study design and study region. Post-hoc investigations on the influence of each individual study on the results of meta-analyses were also performed. |
| √ | Description of statistical methods in sufficient detail to be replicated | The statistics used are described in the methods section. |
| √ | Provision of appropriate tables and graphics | We have provided the following:  Figure 1: Trial Flow Diagram.  Figure 2: Forest plot of random effects meta-analysis with subgroups according to HCC screening. Overall annual HCC incidence.  Figure 3: Forest plot of random effects meta-analysis. HCC incidence in cirrhosis.  Figure 4: Forest plot of random effects meta-analysis. Mortality.  Table 1: Bias assessment.  Table 2: Results, HCC incidence and Mortality. Supplementary 2: Characteristics of included studies |
| **Reporting of results should include** | |  |
| √ | Graph summarizing individual study estimates and overall estimate | Figure 2-4 and table 2 |
| √ | Table giving descriptive information for each study included | Supplementary 2 |
| √ | Results of sensitivity testing | Sensitivity and subgroup analyses are described under the results section. |
| √ | Indication of statistical uncertainty of findings | Results are presented as number of events per 100 person-year with 95% confidence intervals. I-square statistics for heterogeneity and Eggers test for funnel plot assymetry are reported. |
| **Reporting of discussion should include** | |  |
| √ | Quantitative assessment of bias | Quantitative assessment of bias are described in the discussion section. |
| √ | Justification for exclusion | Figure one specifies reason for exclusions |
| √ | Assessment of quality of included studies | Quality assessment are described in both the results and the discussion sections. |
| **Reporting of conclusions should include** | |  |
| √ | Consideration of alternative explanations for observed results | Alternative explanations for the observed results have been described in the discussion section |
| √ | Generalization of the conclusions | Generalizations have been described in the discussion section |
| √ | Guidelines for future research | Guidelines for future research have been described in the discussion section |
| √ | Disclosure of funding source | Disclosures and author contributions are reported. |
